# Bacterial Cellulose for Sustainable Food Packaging: Production Pathways, Structural Design, and Functional Modification Strategies

**DOI:** 10.3390/polym17233165

**Published:** 2025-11-28

**Authors:** Ronagul Turganova, Rysgul Tuleyeva, Ayaz Belkozhayev, Nargiz Gizatullina, Gaukhargul Yelemessova, Anel Taubatyrova, Madina Mussalimova, Zhanserik Shynykul, Gaukhar Toleutay

**Affiliations:** 1 Department of Chemical and Biochemical Engineering, Geology and Oil-Gas Business Institute Named After K. Turyssov, Satbayev University, Almaty 050043, Kazakhstan; ronagul.turganova@gmail.com (R.T.); rysgultuleyeva@gmail.com (R.T.); a.belkozhayev@satbayev.university (A.B.); gizatullinanargiz.nz@gmail.com (N.G.); gauhargul1997@gmail.com (G.Y.); 0ani.lani9@gmail.com (A.T.); mussalimova.m@stud.satbayev.university (M.M.); 2 Research Institute of Advanced Materials, Almaty 040000, Kazakhstan; 3 L2A, INRAE-USC 0340, ENSAIA Department, Faculty of Science and Technology, University of Lorraine, F-54000 Nancy, France; 4 Department of Chemistry, University of Tennessee, Knoxville, TN 37996, USA

**Keywords:** agro-waste, bacterial cellulose, biopolymer, fermentation, sustainable packaging, in situ and ex situ modification, biopolymer, food preservation, bioeconomy

## Abstract

Global concern over food waste and plastic pollution highlights the urgent need for sustainable, high-performance materials that can replace petroleum-based plastics. Bacterial cellulose (BC), a biopolymer synthesized through microbial fermentation by *Komagataeibacter* and related genera, shows exceptional purity, mechanical strength, biodegradability, and structural tunability. Following PRISMA principles, this review analyzed studies from PubMed, Scopus, and Web of Science covering the period 1960–November 2025. Search terms included “bacterial cellulose”, “*Komagataeibacter*”, “*Gluconacetobacter*”, “static culture”, “agitated culture”, “in situ modification”, “ex situ modification”, “fermentation”, and “food packaging”. Inclusion and exclusion criteria ensured that only relevant and high-quality publications were considered. The article summarizes major developments in BC biosynthesis, structural organization, and modification approaches that enhance mechanical, barrier, antioxidant, and antimicrobial properties for food packaging. Recent advances in in situ and ex situ functionalization are discussed together with progress achieved through synthetic biology, green chemistry, and material engineering. Evidence shows that BC-based composites can reduce oxygen and moisture permeability, strengthen films, and prolong food shelf life while maintaining biodegradability. Remaining challenges such as high cost, lengthy fermentation, and regulatory uncertainty require coordinated strategies focused on metabolic optimization, circular bioeconomy integration, and standardized safety frameworks to unlock BC’s full industrial potential.

## 1. Introduction

Currently, the dependency on petroleum-derived plastics within packaging systems is primarily attributable to their exceptional barrier characteristics, technological adaptability, affordability, and production scalability [1]. This worldwide reliance has already culminated in a surge in production volumes, which ascended to 413.8 Mt (million tonnes) in 2023, thereby corroborating forecasts that by 2040, the production and processing of plastics could constitute as much as 20% of global oil consumption [2,3]. It is imperative to acknowledge that such reliance incurs significant environmental ramifications, as a considerable fraction of plastic waste is either incinerated or released without regulation into the environment, inflicting severe harm on ecosystems and posing considerable threats to public health [4]. For instance, it is extensively documented that numerous types of packaging plastics incorporate plasticizers and stabilizers, including phthalates and bisphenol A [5,6]. Over time, these substances have been shown to migrate into food and beverages, which has been associated with endocrine disruption, reproductive health issues, and an elevated risk of oncological disorders [5].

**Figure 1:**
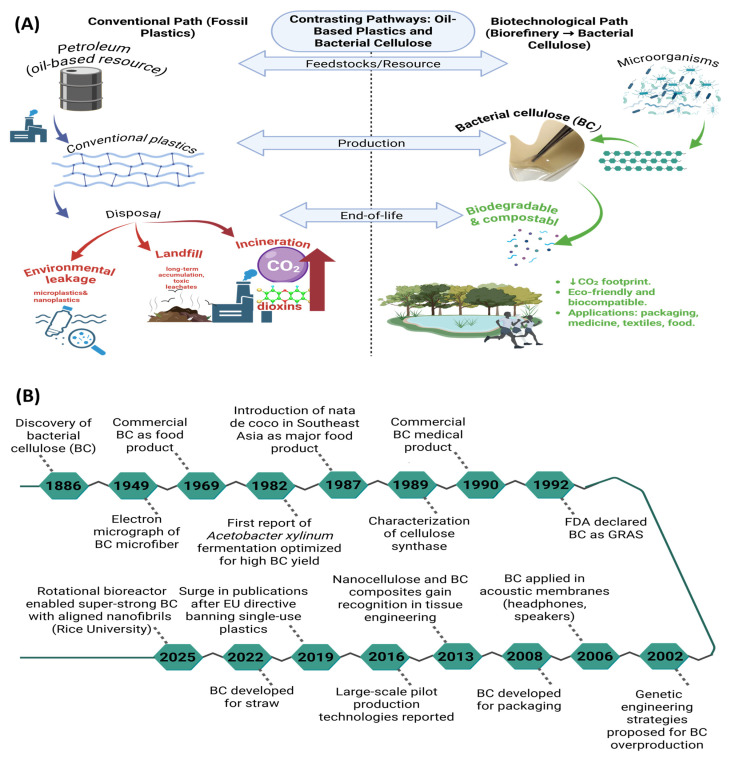
Comparison between the conventional petroleum-based plastic production pathway and the biotechnological route for BC synthesis (**A**) [7,8,9], and historical timeline of major milestones in BC discovery, production optimization, and industrial application from 1886 to 2025 (**B**) [10,11,12,13,14,15].

The pressing necessity to alleviate environmental impact and diminish health-associated hazards propels the exploration of renewable, low-carbon, and potentially carbon-neutral materials [7]. Within the plethora of candidates, cellulose is particularly notable due to its natural prevalence, carbon neutrality, and ecological suitability (Figure 1a) [8,9]. Beyond its origin from plant sources, cellulose can also be biosynthesized by microorganisms as a defensive metabolic strategy, resulting in the formation of bacterial cellulose (BC) [9]. BC was first identified in the late nineteenth century, yet its significance as a versatile biomaterial unfolded gradually through key scientific and technological advances (Figure 1b) [10,11,12,13,14,15]. Early visualization of its unique nanofibrous architecture and later insights into cellulose synthase activity established the foundation for understanding BC biosynthesis [11]. By the late twentieth century, BC had already entered commercial use in both food products and biomedical applications, demonstrating its safety and biocompatibility [12]. The following decades expanded its scope: genetic engineering approaches were proposed to enhance microbial productivity, and applications diversified from acoustic membranes to sustainable packaging solutions [13]. More recently, regulatory and environmental drivers, such as the European Parliament’s directive restricting single-use plastics, have further accelerated interest in BC as an eco-friendly alternative to petroleum-derived polymers [14,15]. These milestones collectively highlight BC’s progression from a biological curiosity to a promising platform for next-generation sustainable materials.

Recent advancements in biotechnology, material science, and sustainable engineering have significantly expanded the potential applications of BC beyond its conventional biomedical and food uses [15]. The integration of synthetic biology, green chemistry, and nanotechnology has facilitated the development of multifunctional BC-based composites with adjustable mechanical, optical, and antimicrobial characteristics [16]. These advances align with a transformative shift in packaging science, moving from passive containment to active, intelligent, and biodegradable systems that preserve food quality while mitigating environmental impacts [17]. In this context, BC has emerged as a crucial platform for the next generation of high-performance, environmentally sustainable packaging materials [18].

Against this backdrop, and in contrast to previous reviews that addressed bacterial cellulose either from a bioprocessing perspective [19] or from a materials engineering standpoint [18,20], the present work provides an integrative framework linking the genetic and biochemical foundations of BC biosynthesis with its structural hierarchy, modification chemistry, and packaging functionality. By systematically distinguishing in situ (modified-medium, aerosol-assisted, and 3D biofabrication) from ex situ (impregnation, casting, vacuum filtration, and electrospinning) strategies, this review develops a multiscale understanding of how each route modulates nanostructure, mechanical reinforcement, and active or intelligent behavior in food-packaging systems. Moreover, unlike bibliometric or single-domain analyses [21,22], this article couples bioprocess optimization, metabolic engineering, and circular bioeconomy concepts to delineate practical pathways toward cost-efficient, low-carbon BC bioplastics. Collectively, it consolidates dispersed advances into a coherent perspective that bridges microbial synthesis, functional modification, and application-oriented performance, defining the foundations for industrial translation of BC-based sustainable packaging.

The aim of this review is to deliver a focused and methodologically transparent synthesis of recent advances in bacterial cellulose production and its translation to sustainable food packaging, and to make clear why an updated analysis is needed now. Unlike prior narratives that treated bioprocessing or materials design in isolation, we connect microbial genetics and fermentation platforms to structural hierarchy and packaging performance, with specific attention to emerging routes such as aerosol-assisted biosynthesis and three-dimensional biofabrication. We map how feedstock choices, reactor modes, and strain engineering in *Komagataeibacter xylinus* and related genera shape fibril architecture and crystallinity, then show how in situ and ex situ modification pathways deliver mechanical reinforcement, barrier control, antioxidant function, antimicrobial efficacy including synergy concepts, and intelligent sensing. We clarify that native bacterial cellulose suffers from specific limitations such as insufficient moisture resistance, restricted flexibility, and modest gas barrier performance, which necessitate the modification strategies discussed throughout the review. We also integrate cross-cutting dimensions that are often fragmented across the literature, including circular bioeconomy substrates, passive radiative cooling films, and the regulatory and techno-economic constraints that govern industrial adoption. By articulating these connections and gaps, the review explains where BC packaging performs well, where it fails, and which process or chemistry levers are most likely to close the remaining cost, durability, and compliance barriers.

## 2. Materials and Methods

Following PRISMA guidance, we searched PubMed, Web of Science Core Collection, and Scopus for records from January 1960 to November 2025. The search combined controlled vocabulary and free-text terms. Core keywords included: “bacterial cellulose”, “BC”, “*Komagataeibacter*”, “*Gluconacetobacter*”, “static culture”, “agitated culture”, “stirred-tank”, “bioreactor”, “Hestrin–Schramm”, “agro-waste”, “glycerol”, “corn steep liquor”, “in situ modification”, “ex situ modification”, “impregnation”, “casting”, “vacuum filtration”, “electrospinning”, “mechanical properties”, “oxygen transmission rate”, “water vapor transmission rate”, “antimicrobial”, “antioxidant”, “intelligent packaging”, and “food packaging”.

Inclusion criteria were: peer-reviewed English articles that focused on bacterial cellulose; reported bioproduction or fermentation parameters; and/or provided structural, physicochemical, or packaging-relevant performance data. Study types included original research, systematic reviews, and high-quality tutorials with experimental details. Exclusion criteria were: non-BCs without separable BC data; conference abstracts without full texts; theses, patents, and editorials; non-English unless uniquely informative; and duplicate or retracted items. From the eligible studies we extracted strain and culture mode, vessel or bioreactor, media and carbon or nitrogen sources, process conditions, yields, crystallinity or degree of polymerization when available, morphology, purification, modification route and technique, composition, and packaging metrics such as tensile properties, oxygen and water vapor transmission, antimicrobial or antioxidant activity, and shelf-life outcomes.

## 3. Bioproduction and Fermentation Strategies of BC

Among all microorganisms capable of synthesizing cellulose, which includes both fungal and algal species, bacteria have been recognized as the most adept and controllable producers, with *K. xylinus* distinguished as the model organism [23]. This superiority can be explained by its distinct microbiological and genetic properties [24]. From a microbiological standpoint, *K. xylinus* is classified as a Gram-negative obligate aerobe exhibiting a highly efficient central metabolic pathway, which enables the ongoing production of cellulose from a wide variety of carbon and nitrogen sources [11]. Genetically, its genome is notable for containing multiple copies of the *bcs* operon, which encodes the cellulose synthase complex (*bcsA*, *bcsB*) along with auxiliary proteins (*bcsC*, *bcsD*) that facilitate the export and crystallization of *β* 1,4 glucan chains, thereby promoting robust fibril formation [11,25]. Physiologically, *K. xylinus* is characterized by its stability, as strains can be maintained 1t low temperatures while preserving their cellulose-synthesizing abilities [11]. Collectively, these characteristics confer exceptional metabolic efficiency, genetic specialization, and storage stability, thereby establishing *K. xylinus* as the most thoroughly documented and extensively employed bacterial species for cellulose production across a range of applications in the fields of food, medicine, and sustainable packaging.

The conventional Hestrin Schramm (HS) medium persists as the standard for bacterial cellulose fermentation within laboratory contexts; nevertheless, its economic feasibility is limited because glucose accounts for nearly 65 percent of total production costs [26]. Consequently, scholars have investigated a wide variety of agro-industrial byproducts as alternative feedstocks, including fruit pomace, tobacco stalks, cotton waste, sugarcane bagasse, dairy whey, brewery by-products, and crude glycerol (Gly) obtained from biodiesel production [19,20,21,22]. These residues are nutritionally useful because they naturally contain the primary metabolites required for *Komagataeibacter* growth and cellulose biosynthesis. Fruit and sugarcane residues supply readily fermentable sugars, dairy whey contributes nitrogen and lactose, and crude Gly or brewery waste provides reduced carbon sources that support efficient energy generation and metabolic flux through cellulose-producing pathways [18,25,26]. Hence, the selection of substrates reflects both the biochemical composition of the waste and its compatibility with microbial metabolism in *Komagataeibacter* [21,22,26]. Additionally, the valorization of these residues promotes fermentation processes that address disposal challenges and lower environmental burdens, which aligns with the principles of the circular economy by converting low-value waste streams into high-value biomaterials [27,28].

The biosynthetic mechanism of bacterial cellulose occurs through two intricately synchronized phases, namely the intracellular polymerization of UDP glucose and the extracellular hierarchical assembly of fibrils; this dual process culminates in a remarkably organized nanostructure that differentiates it from cellulose obtained through plant extraction or chemical regeneration [11]. Specifically, the secreted subfibrils autonomously coalesce into nanofibrils, nanoribbons, and ultimately construct a three-dimensional porous network characterized by high crystallinity and consistent orientation [29]. In contrast, cellulose derived from plants is embedded within a lignocellulosic matrix that comprises lignin, hemicellulose, and pectin, necessitating rigorous chemical and thermal treatments that compromise structural integrity and diminish purity [30]. Similarly, regenerated cellulose, which is synthesized through chemical dissolution followed by reprecipitation, lacks the same nanoscale alignment, resulting in reduced crystallinity and inferior mechanical properties [31]. Consequently, the biosynthetic route of BC endows it with a defect-free nanofibrillar framework exhibiting superior structural alignment and moisture affinity, thereby outperforming plant-derived celluloses in both integrity and interfacial functionality [32,33,34].

Two main fermentation strategies for bacterial cellulose production are static and agitated cultures. In static culture, cellulose forms continuous films at the air–liquid interface under aerobic conditions, yielding highly crystalline and uniform materials suitable for food packaging and biomedical use [35]. Conversely, agitated culture produces irregular morphologies such as pellets and aggregates, and although agitation improves oxygen transfer, it also redirects metabolism toward complete oxidation in the tricarboxylic acid cycle, which reduces cellulose yield [36,37]. Consequently, when evaluating these methodologies, static culture demonstrates superior productivity and structural quality, whereas agitated culture may only be beneficial in particular bioprocess designs that necessitate diverse morphologies. The key differences between these two strategies, including microbial strain, culture type, yield, fermentation duration, and product crystallinity, are summarized in Table 1, which emphasizes their relative industrial advantages and limitations.

Another critical distinction lies in the purification process. Plant derived cellulose is tightly associated with lignin, hemicellulose, and pectin within the lignocellulosic matrix, and therefore its extraction requires high temperatures, aggressive chemical reagents, and energy intensive treatments that compromise both sustainability and cost effectiveness [38]. In contrast, bacterial cellulose is secreted in a nearly pure form, and its purification can be accomplished simply by washing with deionized water to remove residual bacterial cells and medium components [11,24,25,26]. As a result, BC demonstrates a clear environmental advantage, since its recovery minimizes chemical usage, reduces energy consumption, and aligns with eco-friendly bioprocessing principles [22,23,24].

**Figure 2:**
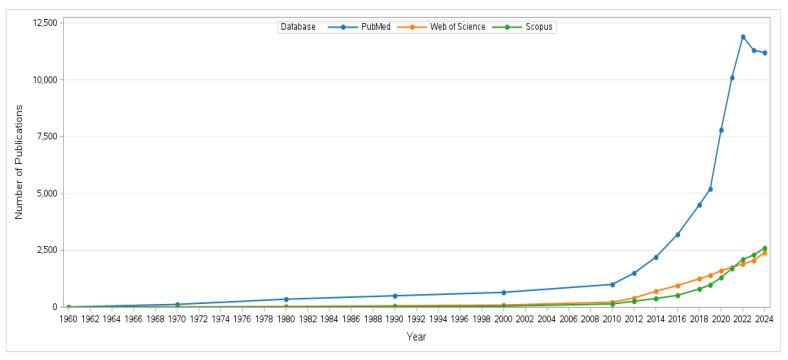
Annual publications on bacterial cellulose from 1960 to 2024, based on searches conducted in Web of Science™, Scopus, and PubMed using the term “bacterial cellulose”.

Analysis of publication trends (Figure 2) reveals a gradual increase in bacterial cellulose research from 1960 onward, followed by a dramatic rise after 2019. This acceleration is closely linked to policy and market pressures within the food-packaging sector, particularly the European Union directive restricting single-use plastics and the global demand for renewable materials with lower environmental impact [2,3,39,40]. For instance, the expansion of the bioeconomy agenda, the introduction of international funding initiatives supporting green materials, and the development of advanced nanotechnology platforms that highlighted BC’s biomedical and packaging potential collectively accelerated scientific attention [39]. Moreover, the COVID-19 pandemic emphasized the demand for safer and biocompatible materials in medical applications, which indirectly stimulated research in microbial cellulose [40]. Collectively, these drivers clarify why BC has become a rapidly growing focus in sustainable food-packaging research, not solely biomedical materials.

Although BC exhibits superior properties, its large-scale production remains limited due to high costs and relatively low yields [41,42]. The standard HS medium alone can account for nearly 30% of total expenses [43], and typical yields rarely exceed 20 g/L, which is insufficient for industrial applications. Static culture offers high-quality pellicles but is constrained by oxygen diffusion and extended incubation periods that hinder scalability. To overcome these limitations, researchers have implemented bioreactor systems, fed-batch fermentation, and statistical optimization through response surface methodology. Bioreactors allow precise control of temperature, pH, and oxygen levels but often fail to reproduce the microstructure obtained under static conditions [41]. Fed-batch and intermittent feeding techniques maintain nutrient availability, enhancing productivity [44]. Further optimization of temperature, pH, and oxygen conditions has been achieved using response surface methodology [45]. Recent efforts have also emphasized replacing conventional nutrients with cost-effective alternatives [21]. Emerging technologies such as cell-free gene expression systems utilize bacterial extracts instead of live cells, offering new opportunities to circumvent the limitations of traditional fermentation and potentially improve process efficiency and scalability [46,47]. Ongoing research is directed toward improving production platforms, refining fermentation conditions, and applying genetic and metabolic engineering strategies to maximize BC yield while minimizing cost [25,48]. In this context, laboratory-scale advances provide valuable insights into improving BC biosynthesis, but they primarily reflect optimizations within controlled experimental settings rather than evidence of broader application trends or market-driven expansion.

**Figure 3:**
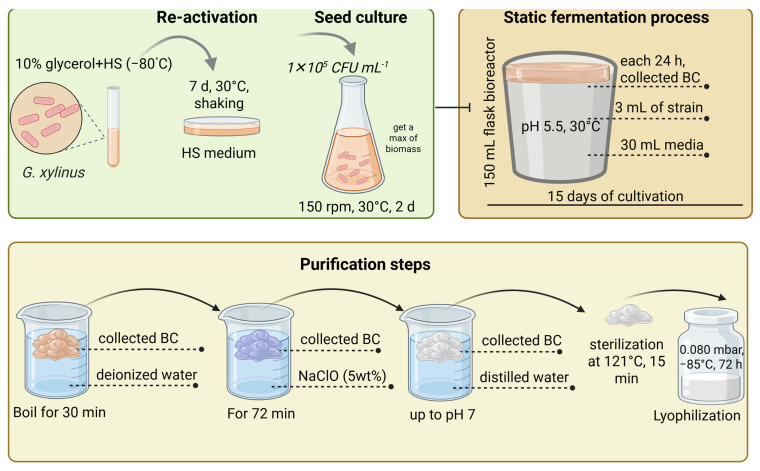
Schematic view of cultivation, and static fermentation in as an example of *G. xylinus* strain adopted from material methods of Saavedra-Sanabria et al. [49].

Recent laboratory studies have also demonstrated optimized static cultivation approaches, as illustrated below. An example of the static cultivation process was described by Saavedra-Sanabria et al. [49], who cultivated *G. xylinus* strains preserved in 10% Gly at −80 °C. Frozen strains were reactivated in fresh HS medium at 30 °C with shaking for 7 d, followed by preparation of seed cultures in 150 mL HS medium incubated at 150 rpm and 30 °C for 48 h. A suspension containing 1 × 10^5^ CFU mL^−1^ was inoculated into bioreactor flasks containing 30 mL of nutrient medium and 3 mL of seed culture. The mixture was adjusted to pH 5.5 and incubated statically at 30 °C for 15 d. BC pellicles were collected daily, and both pH and sugar consumption were monitored. After fermentation, the BC films were purified by boiling in deionized water for 30 min, followed by immersion in 5% sodium hypochlorite (NaClO) solution for 72 h, washing to neutral pH, sterilization at 121 °C for 15 min, and lyophilization under vacuum at −87 °C for 72 h (Figure 3).

## 4. Structural Basis of BC

BC demonstrates a distinctive semicrystalline architecture that comprises both organized crystalline domains and disordered amorphous regions. In the crystalline domains, *β*-1,4-glucan chains assume a highly ordered parallel configuration, which is stabilized by extensive networks of intra- and inter-chain hydrogen bonding, with each anhydroglucose unit contributing three reactive hydroxyl groups (C2, C3, C6). In contrast, the amorphous regions contain a greater abundance of free hydroxyl groups, which confer flexibility and hydrophilicity. Quantitative assessments have indicated that the crystallinity index of BC (84–90%) significantly surpasses that of cellulose derived from plants (40–60%), attributable to the superior enzymatic precision exhibited by cellulose synthase complexes during the biosynthetic process [50]. The crystalline architecture of cellulose presents various polymorphic forms (I–V) that are contingent upon the orientation of hydrogen bonds within and between the chains. Despite both BC and plant cellulose exhibiting the cellulose I structure characterized by parallel-chain packing, their supramolecular organizations manifest substantial differences [51,52,53]. The three-dimensional network of BC is constituted of nanofibers interconnected via intramolecular and intermolecular hydrogen bonding, culminating in a high specific surface area (50–200 m^2^ g^−1^) and porosity exceeding 90% [54,55]. These microstructural attributes endow BC films with exceptional strength, elasticity, and reactivity in comparison to conventional plant cellulose.

Cellulose typically exists as a mixture of two crystalline allomorphs, namely Iα (triclinic) and I*β* (monoclinic) [51,56]. The Iα phase is predominantly found in BC and certain algal celluloses, while the I*β* phase is more characteristic of celluloses derived from plants [56,57]. These structural features and their distribution across bacterial and plant celluloses have long been recognized in classical cellulose science and form the basis for distinguishing microbial BC from lignocellulosic counterparts. For instance, *Gluconacetobacter hansenii* NCIM 2529 yields a BC with a crystallinity reaching up to 81%, a predominant I*α* phase, a Z-average particle size of 1.4 µm, and a porosity of 182% [57]. The degree of polymerization (DP) for BC typically ranges from 2000 to 6000 glucose units, whereas plant cellulose exhibits significantly higher DPs (13,000–14,000) [58]. Variations in DP and microstructure as a function of cultivation conditions have also been well established in foundational BC studies. Under static culture at pH 4, *Acetobacter xylinum* synthesized BC with DP values of 14,000–16,000, whereas a moderate elevation to pH 5 diminished it to approximately 11,000 [59]. These observations reflect classical experimental procedures widely used to investigate how pH, oxygen availability, and nutrient composition affect BC polymerization and microfibril assembly, rather than representing newly developed methods. These variations underscore the intricate relationship between cultivation parameters and the molecular architecture of BC.

**Figure 4:**
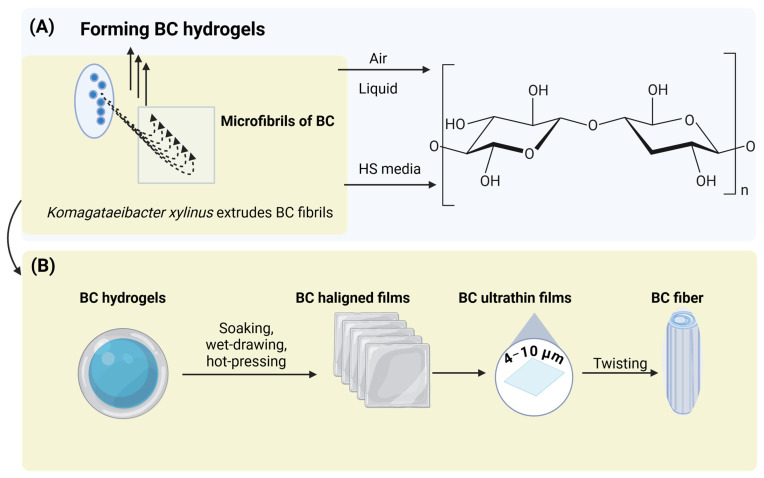
(**A**) Segment of the hypothetical synthetized BC [55,61]; (**B**) BC hydrogels, films and fiber formations adopted from Wu et al. [70].

BC films manifest a stratified three-dimensional nanostructure consisting of robust fibrils (150–160 nm) organized from diminutive nanofibrils (20–60 nm) that are formed from *β*-1,4-glucan chains [54,60] (Figure 4a). This intricate network bestows remarkable mechanical durability and contributes significantly to the high water-holding capacity (WHC) of BC [61,62]. The pores within BC range from nanometer to micrometer scale, with average mesopores measuring 21–26 nm and pore volumes between 0.024 and 0.11 cm^3^ g^−1^ [63]. Typically, BC gels demonstrate WHC values ranging from 62.3 g·g^−1^ in their hydrated state to 3.8 g·g^−1^ post-drying [60,64]. On a dry-weight basis, newly synthesized BC possesses the capability to retain water at a ratio of 62 to over 100 times its weight, which corresponds to approximately 98.8% moisture content [65,66]. This extraordinary WHC is attributable to its three-dimensional nanostructure, extensive surface area, and the presence of numerous hydroxyl groups that facilitate the formation of hydrogen bonds [67]. Various environmental factors also play a significant role in influencing WHC: *K. hansenii* GA2016 cultivated on a fruit-peel medium exhibited a WHC of 627–928%, surpassing the 609% observed in HS medium, with fibril diameters ranging from 47 to 61 nm [68]. In a similar vein, *Novacetimonas hansenii* P3 cultivated on pomegranate waste exhibited a WHC of 554% alongside fibril widths of 50–70 nm [69]. Water functions as a plasticizer, thereby augmenting flexibility and permeability (Figure 4b) [67,68,69,70].

The production of BC is profoundly affected by the selection of carbon sources, which can constitute 30–65% of the total production expenses [71,72]. Frequently employed substrates encompass glucose, sucrose, fructose, and mannitol [73,74]. Glucose, recognized as the conventional carbon source for *A. xylinum*, often results in the accumulation of gluconic acid, which subsequently lowers the pH of the culture medium and inhibits BC biosynthesis [75]. Alternative carbon sources such as fructose and Gly can yield comparable outcomes; however, monosaccharides like galactose and xylose typically result in diminished productivity due to the slower growth rates of the bacteria. Notably, certain alternatives have exhibited enhanced efficiency; for example, D-arabitol has demonstrated a BC yield exceeding six times that of glucose in *A. xylinum* KU-1 [75,76].

Despite its high yield, the cost-effectiveness of D-arabitol remains a limiting factor for large-scale BC production. Unlike glucose, which is produced on an industrial scale through starch hydrolysis and enzymatic saccharification, D-arabitol is primarily obtained from microbial fermentation of sugars such as D-glucose or D-xylose using *Candida* and *Debaryomyces* species. This additional bioconversion step increases production costs, making D-arabitol less economically competitive compared with conventional carbon sources. According to current market analyses, the price of D-arabitol remains several times higher than that of food-grade glucose, thereby constraining its feasibility for continuous fermentation systems where carbon source input strongly influences cost per gram of cellulose yield [19,20,21,22].

However, ongoing research seeks to improve its market accessibility by employing renewable feedstocks and engineered microbial routes for D-arabitol biosynthesis. For instance, lignocellulosic biomass hydrolysates and agricultural residues have been explored as inexpensive substrates for D-arabitol production, significantly lowering process costs while supporting circular bioeconomy principles. Thus, while D-arabitol demonstrates remarkable bioconversion efficiency and compatibility with BC-producing strains, its large-scale adoption will depend on future advances in low-cost biomanufacturing and supply chain availability that can match the affordability of glucose [20,21,22].

To reduce production costs, recent studies categorize alternative feedstocks for *Komagataeibacter* fermentation into three main groups of carbon sources. The first group includes fruit-derived substrates, such as pineapple peel juice, overripe fruit, and other sugar-rich residues, which provide readily fermentable monosaccharides and support high cellulose yields [77]. The second group comprises agricultural wastes, including sugarcane juice, wheat straw hydrolysates, and coffee husks. These materials contain fermentable sugars and organic acids that can sustain microbial growth after appropriate pretreatment [21]. The third group encompasses industrial by-products, most notably crude glycerol (Gly), corn steep liquor, fermentation residues from wine and beer, and cotton textile waste. These substrates offer reduced carbon compounds and nitrogen-rich components that enhance microbial metabolism and biomass formation [21,77]. The choice of carbon source not only affects productivity but also modulates the morphology and organization of BC fibrils. For example, BC synthesized from xylose forms less uniform microfibrils than cellulose produced from glucose [75]. *A. xylinum* can metabolize a wide variety of these substrates through pathways such as the pentose phosphate pathway, the tricarboxylic acid cycle, and gluconeogenesis, achieving an approximate carbon-to-cellulose conversion efficiency of 50 percent [78].

Nitrogen sources are also integral to the biosynthesis of BC. Conventional nitrogen supplements, including yeast extract and peptone, are costly and may account for 50–65% of the overall production costs [79,80,81]. To overcome this challenge, alternative, lower-cost nitrogen sources derived from waste materials have been investigated. A particularly effective substitute is corn steep liquor, a by-product of the starch industry [82]. Additionally, coffee cherry husk extract has shown promise as a nutrient source, while sunflower meal hydrolysates, in combination with crude Gly and hydrolysates from confectionery waste, have each yielded approximately 13 g/L of BC [83,84].

Several additives are known to enhance BC yield and improve material properties. Ethanol, vitamins, agar, sodium alginate, sulfates, and phosphates have been frequently tested. Ethanol not only increases yield but also minimizes the occurrence of non-producing mutant strains in agitated cultures [85]. Volova et al. [86] observed that the addition of 3% ethanol to a glucose–Gly medium elevated BC production in *K. xylinus* B-12068 to 2.4–3.3 g L^−1^ d^−1^. Vitamin additives can also promote productivity; for example, supplementation with 0.5% ascorbic acid doubled both BC yield and crystallinity in four *Gluconacetobacter xylinus* strains [87]. Modification of medium rheology using agar or sodium alginate, along with mineral additives such as sulfates and phosphates, has proven beneficial for improving BC synthesis [85,88]. In parallel, the selection of an appropriate carbon source must reflect realistic industrial availability; although pure glucose is frequently used in laboratory media, industrial processes increasingly rely on hydrolysates obtained from sugar-rich, starchy, or lignocellulosic feedstocks to reduce cost and ensure resource sustainability. Accordingly, optimization of carbon and nitrogen inputs must account for both biological performance and the techno-economic feasibility of sourcing hydrolysates rather than importing refined glucose. Current research focuses on cost-effective medium formulations and the integration of synthetic biology and metabolic engineering approaches for targeted pathway enhancement [85,89].

Building upon the previously described fermentation strategies, the operational parameters of static cultivation have been extensively optimized to maximize BC yield and crystallinity. Under typical conditions, cultures are maintained in shallow trays or flasks at 28–30 °C and pH 4–7 for 1–14 d without agitation. During fermentation, a dense pellicle develops at the air–liquid interface, where restricted oxygen diffusion often becomes a rate-limiting factor [90,91,92]. To enhance productivity, several studies have explored temperature control and carbon-source variation. For instance, Sathianathan et al. [69] isolated *N. hansenii* P3 from decayed pomegranate fruit waste and achieved a yield of up to 3 g L^−1^ of BC with a crystallinity index of 96% when cultivated in HS medium containing glucose and sucrose at 30 °C for 15 d.

Food-grade and waste-derived carbon sources can be categorized into three principal groups based on their carbohydrate composition and required processing steps: (i) sugar-containing materials (e.g., fruit juices, molasses, cane or beet syrups), which can be used directly after dilution; (ii) starchy materials (e.g., potato waste, cassava residues, corn by-products), which require enzymatic or acid hydrolysis to release fermentable sugars; (iii) cellulose-containing plant biomass (e.g., agricultural residues, fruit peels, brewery waste), which must undergo pretreatment and saccharification to generate glucose-rich hydrolysates. Such a classification enables a clearer evaluation of technological routes for converting inexpensive feedstocks into fermentable media suitable for BC production and highlights the importance of matching substrate type with appropriate processing intensity.

In contrast, agitated cultivation involves continuous movement of the culture medium through shaking or stirring [93,94]. Under these conditions, BC forms as irregular particles or spherical pellets dispersed throughout the liquid rather than as a uniform film [95,96]. The morphology depends on rotational speed: spherical aggregates typically form above 100 rpm, while irregular forms appear at lower speeds. Agitation enhances oxygen availability, accelerates cellulose synthesis, and generally yields higher productivity compared to static culture [41,97]. However, BC obtained from agitated fermentation exhibits lower crystallinity, shorter polymer chains, and reduced mechanical strength [90,98]. Moreover, continuous agitation may induce mutations leading to non-cellulose-producing strains, ultimately decreasing yield [96]. Despite these drawbacks, agitated culture offers advantages for large-scale manufacturing by reducing production time by up to 90%, increasing yield, and simplifying process scalability. Therefore, most commercial BC is produced using agitated fermentation when uniform film morphology is not required [93].

Crystallinity exerts a significant influence on pore architecture and mechanical stability, demonstrating an inverse relationship: elevated crystallinity corresponds to a reduction in porosity and swelling [99,100]. Additives such as calcofluor (CF) and carboxymethyl cellulose (CMC) modify the morphology of microfibrils, resulting in a reduction in width from 65 nm (untreated) to 32 nm (CMC) and 49 nm (CF), simultaneously decreasing crystallinity from 85% to 71% and 55%, respectively [101]. Alkaline post-treatment similarly impacts these properties. Films derived from *A. xylinum* exhibit tensile strength up to 208 MPa; however, treatment with 10% NaOH induces fibril swelling and fracture, resulting in a decrease in strength to 162 MPa [102]. Conversely, optimized purification employing 0.01 M NaOH at 70 °C for 2 h maintains structural integrity [103]. Composite BC materials (such as BC–acrylic acid hydrogels) display pore diameters ranging from 10 to 100 µm and mesh sizes approximately 3 nm [104]. These hierarchical configurations promote enhanced permeability and stability, thereby extending the applicability of BC to areas such as wound dressings and packaging [105].

BC demonstrates exceptional thermal and mechanical stability when juxtaposed with traditional petroleum-based plastics [62]. The tensile strength is observed to range between 18 and 22 MPa, accompanied by a Young’s modulus of 15–18 GPa [47,106]. Following wet-drawing and hot-pressing processes, ultrathin BC films (4–10 µm) derived from *G. xylinus* achieve a strength of 758 MPa and a toughness of 42 MJ m^−3^; twisted fibers attain 954 MPa and 93 MJ m^−3^ [71,107]. The thermal degradation pathway occurs through dehydration, depolymerization, and glycosidic bond cleavage, with T_dmax ranging from 319 to 374 °C and a minimal weight loss occurring below 100 °C [104,108]. Dynamic mechanical analysis indicates that BC sustains a stable storage modulus within the temperature range of −130 °C to 200 °C, in contrast to petroleum-based plastics, which exhibit degradation of modulus as a function of temperature [109,110]. These observations reflect intrinsic material properties of BC and do not depend on the use of degraded or spoiled raw materials for fermentation. Rather, BC’s superior stiffness and thermal resilience arise from its highly crystalline nanofibrillar architecture, regardless of whether the carbon source is a refined sugar or a hydrolysate-based substrate.

In addition to its mechanical robustness, BC provides remarkable biocompatibility and environmental sustainability [111,112]. It exhibits chemical stability, resistance to ultraviolet light, and thermal durability up to 250 °C [112]. The absence of lignin and hemicellulose in BC facilitates its chemical functionalization [113]. Furthermore, the material is non-toxic and elicits a minimal immune response, thereby ensuring safety for biomedical applications [112]. BC membranes are characterized by transparency and biodegradability in soil within 2 to 9 weeks [47,60], presenting a feasible alternative to address microplastic pollution [27,28,29,30,31]. The incorporation of natural antimicrobials (such as chitosan, carvacrol, silver nanoparticles, or plant oils) augments antibacterial efficacy against *E. coli* and *S. aureus* [114]. The blending of BC with other biopolymers (e.g., gelatin, alginate, lignin) enhances both mechanical and barrier properties [113]. In comparison to petroleum-based plastics (such as polyamide, polycarbonate, polyoxymethylene, polypropylene), which soften and deform at approximately 200 °C, BC-based materials maintain structural integrity and biodegrade within roughly 45 days [60,72].

## 5. BC Production and Modification Strategies for BC

### 5.1. Overview of Production and Modification Approaches

BC inherently exhibits a finely organized microfibrillar network that confers outstanding tensile resilience, thermal endurance, and environmental degradability, positioning it as a competitive substrate for sustainable plastic alternatives. Despite its robust crystalline architecture, native BC presents several limitations, including insufficient flexibility, high moisture sensitivity, and limited surface functionality, which can restrict its performance in packaging applications. As a result, structural modification and hybridization methodologies have become essential to address these deficiencies. The plentiful hydroxyl groups on the BC backbone offer multiple reaction sites for targeted chemical functionalization. Through classical surface-modification techniques such as phosphorylation, acetylation, and benzoylation, properties including hydrophobicity, thermal stability, and interfacial compatibility can be systematically adjusted. These chemical modifications strengthen intermolecular interactions within composite matrices, thereby improving mechanical strength, gas barrier capacity, and thermal resistance [110,115,116,117,118,119,120,121].

Moreover, BC’s three-dimensional nanofibrillar network and high specific surface area enable its use as a reinforcing scaffold for numerous additives. When incorporated into polymer matrices, BC nanofibers enhance structural cohesion, reduce water permeability, and enable uniform dispersion of active agents such as antimicrobials, antioxidants, freshness indicators, radiative modifiers, and ethylene scavengers. This versatility supports the development of multifunctional BC-based packaging systems [119,122,123,124,125,126,127,128,129].

Moreover, the relevance of BC for food packaging is increasingly linked to its availability from kombucha-derived Symbiotic Culture of Bacteria and Yeast (SCOBY), which is already produced at industrial scale. The global kombucha market was estimated at approximately 4.26 billion USD in 2024 and is projected to reach about 9.09 billion USD by 2030, indicating that cellulose-rich SCOBY pellicles are continuously generated as a by-product of large-scale beverage manufacture. Recent bibliometric analyses show that a substantial fraction of SCOBY-related publications focus on bacterial cellulose production, strain selection, and process optimization, reflecting a mature technological base for SCOBY-derived BC [130]. Techno-economic modelling of a kombucha-based BC facility with an annual capacity of 60 t reported a capital investment of around 13.7 million USD, operating costs of about 3.8 million USD per year, a payback time of approximately 4.2 years, and an internal rate of return near 16%, thereby demonstrating that SCOBY-derived BNC can be produced under economically viable conditions [130,131]. These data indicate that modification strategies discussed in this section can build on a realistic industrial manufacturing context rather than purely laboratory-scale routes.

**Figure 5:**
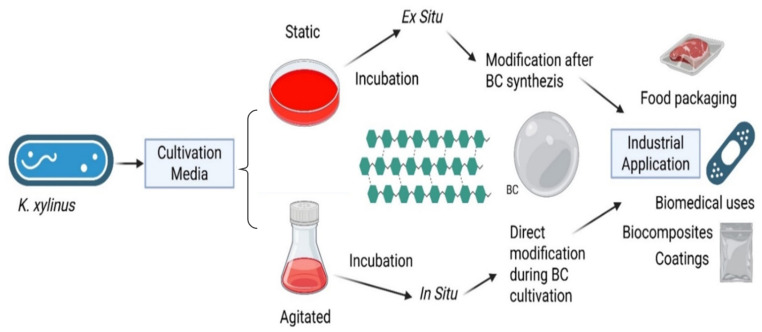
Schematic overview of BC production from *K. xylinus* via static and agitated cultivation and its subsequent in situ and ex situ modifications for industrial applications.

In particular, the incorporation of external compounds before or after biosynthesis results in two primary modification pathways: in situ and ex situ. These pathways are independent of cultivation mode (static or agitated) and can be applied to BC produced through any fermentation system. In situ modification involves adding functional precursors or tuning fermentation conditions so that functional moieties become incorporated during cellulose biosynthesis. By contrast, ex situ modification is performed on purified BC via impregnation, grafting, coating, or other post-synthetic treatments, enabling more controlled and reproducible functionalization (Figure 5)**.** Each method alters BC’s porosity, mechanical resilience, and surface chemistry in distinct ways; thus, the choice of pathway depends on the intended application. Together, these strategies facilitate the creation of active BC-based films with enhanced mechanical, barrier, and antimicrobial properties, supporting recent advances in multifunctional food-packaging materials (Table 2) [117,118,119,120,121,122,123,124,125,126,127,128,129].

Given the growing interest in BC as a replacement for petroleum-based packaging materials, a quantitative comparison of its mechanical and thermal performance with conventional plastics is essential to contextualize its advantages and limitations (Table 3).

### 5.2. In Situ Method

In situ modification preserves the fundamental structural integrity of BC by introducing functional components directly during biosynthesis rather than through post-processing. Three rigorously validated methodologies are commonly applied, each differing in how additives are incorporated and how fibril assembly is controlled [115,116,117,118,119,120,121,122,123,124]. Modified-medium cultivation introduces polymers, polysaccharides, or bioactive molecules into the culture broth, allowing them to co-assemble with cellulose fibrils during secretion and primarily altering crystallinity, flexibility, and hydration behavior. Aerosol-assisted biosynthesis delivers nanoparticles or functional agents to the air–liquid interface, promoting uniform distribution of nano-units and yielding hybrid structures with enhanced mechanical and barrier properties. Three-dimensional biofabrication employs molds, templates, or programmable bioinks to direct bacterial growth spatially, enabling BC with tailored porosity, thickness, and geometric configuration suited for advanced packaging formats. Across these approaches, exogenous components interact with BC nanofibres through hydrogen bonding, electrostatic interactions, or van der Waals forces, thereby modulating the mechanical, optical, and antibacterial characteristics of the resulting materials.

Given the brittleness and constrained flexibility of BC, which restrict its applicability in packaging domains, numerous strategies have been proposed to mitigate these limitations. A particularly effective approach entails the incorporation of water-soluble polymers into the culture medium to enhance flexibility and elongation capacity. For example, Wu et al. [115] introduced high-acyl and low-acyl gellan gums into the fermentation system, yielding flexible BC/high-acyl gellan gum (BC/HA) and BC/low-acyl gellan gum (BC/LA) composite films. The gellan gum was integrated within the three-dimensional BC matrix during the biosynthetic process, thereby disrupting the structural symmetry and regularity of the cellulose chains. In comparison with pure BC, the crystallinity index exhibited a substantial reduction from 89% to 73% and 67% for BC/HA and BC/LA, respectively [115].

Furthermore, the tensile strength of BC can be substantially enhanced by augmenting the fibre diameter. Gao et al. [116] integrated xanthan gum (XG) into the fermentation medium, resulting in BC/XG nanocomposites that displayed a significant augmentation in tensile strength relative to pure BC. This enhancement was ascribed to the interaction and intertwining of XG with the cellulose microfibrils secreted by the bacteria, leading to an increase in the diameter of cellulose nanoribbons during the self-assembly process.

In addition to mechanical strength, antibacterial efficacy constitutes another pivotal attribute for packaging materials, as excessive microbial growth accelerates food spoilage during storage. Chen et al. [117] extracted flavonoids from mulberry leaves and incorporated them into the fermentation medium, yielding BC composites with antibacterial activity surpassing 50% against *E. coli* and *S. aureus.* Nonetheless, this methodology presents two significant limitations. On one hand, the presence of bacteriostatic compounds impedes microbial activity, consequently diminishing fermentation efficiency. On the other hand, purification processes aimed at eliminating residual medium and microbial cells frequently result in the leaching of water-soluble bioactive compounds, thereby leading to a reduction in antibacterial efficacy [117].

To mitigate these challenges, advanced chemical bonding methodologies have been formulated to avert the depletion of active molecular species during the purification process. Liu et al. [118] employed glucose as a molecular carrier by covalently linking fluorescent photosensitizers to it, subsequently incorporating this compound into the fermentation milieu to synthesize functionalized BC exhibiting unique non-natural fluorescence and pronounced antibacterial properties. This methodology significantly reduces the performance degradation of additives throughout purification and signifies a promising avenue for the production of functionalized BC via in situ biosynthetic processes [118].

The aerosol-assisted biosynthesis technique further augments the mechanical and functional properties of BC-based composites by facilitating the uniform distribution of nanoscale entities such as nanospheres, nanowires, or nanosheets at the BC growth interface [119,120,121,122]. In this method, a fine aerosol is generated using atomizing nozzles operating at controlled air pressures (typically 0.1–0.3 MPa) and droplet sizes in the range of 10–50 µm. The aerosol is sprayed intermittently or continuously onto the air–liquid interface of the culture medium under stable temperature and humidity to prevent excessive turbulence. During the biosynthetic process, oxygen, nutrients, bacterial cells, and nano-units engage in dynamic interactions, and the deposited nano-units become intricately entangled with cellulose nanofibers, forming a self-assembled hybrid architecture. The distributed nano-units inhibit fiber aggregation during fermentation and promote finer cellulose morphologies relative to pure BC [119]. As a result, the reduction in fiber diameter enhances both toughness and light transmittance by minimizing scattering. Guan et al. [119] synthesized BC/synthetic mica composites using aerosol-assisted biosynthesis and achieved a toughness of 16 MJ m^−3^, corresponding to a 164 percent improvement over pure BC. Similarly, Zhang et al. [119] incorporated nano-clay into BC with the same method and produced films with approximately 1.5 times greater visible light transmittance than pure BC films.

Another significant advancement pertains to the integration of nano-units endowed with intrinsic cooling characteristics for packaging applications necessitating thermal regulation. The maintenance of a low internal temperature during storage is paramount for the preservation of food products. Passive radiative cooling (PRC) provides an energy-efficient and emission-free solution for this requirement [120]. Given that SiO_2_ nanoparticles exhibit high emissivity within the atmospheric window, Shi et al. [120] assimilated these nanoparticles into the BC matrix through aerosol-assisted biosynthesis to formulate a BC-based radiative cooling film (Bio-RC) with a reflectivity of 95% and infrared emissivity of 93%. Field evaluations indicated that Bio-RC could lower surface temperature by approximately 3.7 °C relative to ambient conditions, thereby demonstrating significant potential for food packaging applications featuring passive cooling capabilities [120].

Moreover, the aerosol-assisted biosynthesis methodology facilitates the controlled incorporation of antimicrobial nanomaterials [119,121]. Since microorganisms at the growth interface are encapsulated within the BC network, the direct infiltration of antimicrobial compounds is constrained; however, this technique permits precise administration and curtails the excessive application of antibacterial agents. Wan et al. [121] successfully engineered BC/AgNW composites with markedly improved inhibition zones (5.8 mm for *E. coli* and 6.5 mm for *S. aureus*) when contrasted with conventional silver nanoparticle-laden (AgNP-laden) BC materials. To counteract turbulence at the liquid–air interface resultant from aerosol impact, Zhang et al. [119] devised a gelling culture medium that stabilized the interface and facilitated the formation of multilayer BC films. The resultant multilayered film exhibited exceptional moisture barrier efficiency (1.76 g·mm·m^−2^·d^−1^·kPa^−1^ at 75% RH), substantial mechanical strength (462 MPa), and antibacterial efficacy exceeding 90%, thereby affirming its potential for multifunctional BC composites characterized by hierarchical structures.

Traditional BC manifests as a thin and dense biofilm under static culture conditions due to the constraints imposed by oxygen diffusion; conversely, three-dimensional (3D) biofabrication affords meticulous spatial regulation over bacterial proliferation, thereby facilitating the generation of materials characterized by adjustable thickness and a porous architecture [122,123,124]. This methodology enhances the formulation of packaging materials endowed with thermal insulation properties, gas adsorption capabilities, and resilience to impact. Laurent et al. [122] pioneered a technique that amalgamates direct ink writing with hydrogel-based bioinks containing microorganisms with high yield and biocompatible polymers. The hydrogel matrix not only fostered bacterial proliferation and metabolic activity but also permitted precise spatial control during the printing process, culminating in the production of 3D BC structures with programmable configurations. In a similar vein, Ajdary et al. [123] employed pre-engineered molds to guide microbial biosynthesis, thereby achieving a high-fidelity replication of intricate design patterns. An alternative methodology encompasses the utilization of solid templates, such as agarose, starch, wax, or gelatin, to induce porosity within BC. Lin et al. [124] adopted a foaming technique that incorporated chitosan to develop porous BC packaging materials that exhibited antibacterial efficacy exceeding 95% against both *E. coli* and *S. aureus.*

### 5.3. Ex Situ Method

BC can be further purified and transformed into diverse structural forms, including BC films, BC nanofibrils (BCNFs), and bacterial cellulose nanocrystals (BCNCs), to enhance its physicochemical and functional properties through ex situ modification techniques. BCNF is typically generated via the mechanical disintegration of BC films, whereas BCNC is derived through acid hydrolysis of BC. In contrast to in situ modification approaches, ex situ methodologies offer increased flexibility in the selection of modifying agents, as there exists no requisite to evaluate their potential effects on bacterial proliferation or cellulose biosynthesis. Importantly, native BNC pellicles obtained directly from static or agitated cultivation can also serve as substrates for ex situ composite formation, as their intact three-dimensional nanofibrillar network readily accommodates post-synthetic impregnation, coating, and particle loading. By integrating these various forms of BC with alternative functional materials via processes such as impregnation, casting, vacuum filtration, or electrospinning, researchers are capable of generating hybrid composites that exhibit superior mechanical, optical, and barrier attributes, thereby effectively addressing the constraints associated with individual constituents. The inherent three-dimensional network structure of BC provides a hierarchically porous scaffold conducive to the anchorage of exogenous functional moieties, while solvent-phase impregnation has emerged as a straightforward yet precise technique for augmenting mechanical strength, optical clarity, and antibacterial efficacy [111,122,123,124,125,126,127,128,129]. For instance, when antibacterial agents are incorporated into BC-based packaging, their selection must also consider potential migration limits and established food-contact safety regulations to avoid unintended impacts on human enzymatic systems and commensal microbiota.

Yang et al. employed the impregnation technique to facilitate the introduction of sodium alginate into BC through osmotic pressure and capillary action [111]. The sodium-alginate-embedded BC was subsequently immersed in a calcium lactate solution, resulting in the formation of BC/calcium-alginate composites wherein calcium alginate functioned as a structural mediator within the three-dimensional nanonetwork, effectively fortifying the cellulose matrix and promoting inter-fibrillar stress distribution. Consequently, the BC/calcium-alginate composite exhibited remarkable tensile strength (420 MPa) and toughness (24 MJ m^−3^). Drawing inspiration from the structural roles of cellulose and lignin in natural wood, Liu et al. integrated lignin into BC and chemically reduced it to alkali lignin (AL) during the impregnation process utilizing acetic acid, thereby developing BC/AL composites characterized by exceptional compressive strength (2.4 MPa) [111].

In addition to providing mechanical reinforcement, the impregnation methodology is capable of modulating the optical properties of BC-based composites. Polyvinyl alcohol (PVA), a hydrophilic polymer extensively utilized in films and coatings, enhances optical transparency by mitigating refractive index discrepancies within the material. Given that UV radiation has the potential to compromise the quality and nutritional integrity of food, the development of packaging materials endowed with UV-shielding capabilities is of paramount importance. Gly, known for its pronounced UV-absorption properties, can be employed to augment UV-blocking efficacy. Cazón et al. synthesized BC–Gly–PVA composites by immersing BC in a mixed PVA–Gly solution, yielding materials with UV absorbance levels of approximately 99% (190–280 nm), 97% (280–320 nm), and 90% (320–400 nm) [60]. Concurrently, Gly and PVA, which are abundant in hydroxyl groups, can enhance hydrogen bonding interactions with water molecules, thereby increasing the water permeability of the resultant films.

Metallic nanoparticles, including those composed of gold, silver, copper, and zinc, exhibit significant antibacterial properties; however, their application is typically constrained by their incompatibility with microbial proliferation during in situ biosynthesis. Consequently, ex situ impregnation emerges as a more secure and manageable methodology for the integration of these nanoparticles into BC matrices. Zhang et al. conducted an experiment in which BC was submerged in a silver nitrate (AgNO_3_) solution, subsequently reducing silver ions to silver nanoparticles via sodium borohydride (NaBH_4_), thereby achieving a homogenous dispersion of nanoparticles throughout the BC framework. The impregnation technique effectively alleviated the aggregation of nanoparticles, a phenomenon exacerbated by nanoconfinement and interfacial interactions, thus presenting a dependable strategy for the production of enduring antibacterial packaging materials [126].

The casting technique embodies an alternative straightforward and economical strategy for the synthesis of BC-based composites, which may include coatings, multilayer films, and hydrogels. In this particular process, BC is amalgamated with functional constituents to generate a slurry, which is subsequently poured into molds and dried under regulated thermal conditions. Within this slurry, BC predominantly manifests as nanofibrils or nanocrystals, which enhance the composite’s structural integrity through hydrogen bonding and nanoscale reinforcement. Chen et al. successfully formulated chitosan/polyvinyl alcohol (PVA)/BC/ginger-essential-oil composite films that exhibited an 87% enhancement in tensile strength in comparison to the original chitosan/PVA films, thus illustrating the reinforcing influence of the highly crystalline BC nanostructures [129]. Additionally, the casting methodology facilitates the incorporation of targeted functional components that bestow antibacterial or antioxidant properties. Li et al. integrated zinc chloride (ZnCl_2_), BCNF, and sodium alginate to fabricate antibacterial hydrogels that demonstrated lethality rates exceeding 95% against both Escherichia coli and Staphylococcus aureus [127].

However, the phenomenon of dispersion instability within BC slurries, attributed to hydrogen bonding and van der Waals forces, can compromise structural uniformity, thereby diminishing the mechanical properties of the resultant composites. To address this challenge, Lin et al. introduced a covalent-conjugation methodology wherein chitosan (CS) was chemically grafted onto BC through Schiff-base reactions, yielding a more homogeneous slurry. The resultant CS–BC composite films demonstrated a remarkable enhancement of 90% in tensile strength (95 MPa) and a 60% increase in elongation when contrasted with untreated CS–BC films [124,125].

Conventional casting methodologies frequently depend on thermal evaporation for solvent removal, a process that may compromise thermolabile additives. While cryogenic lyophilization retains structural integrity, it incurs substantial costs and significant energy expenditure. Conversely, vacuum-assisted filtration offers a rapid solvent extraction mechanism through pressure differentials at ambient temperatures. This technique significantly improves polymer–filler interfacial interactions and promotes the development of homogeneous composite films. Due to its inherent flexibility and porous architecture, BC is exceptionally well-suited for applications as surface-enhanced Raman scattering (SERS) substrates. Zhang et al. synthesized Ag-nanorod-decorated BC (AgNRs@BC) sensors utilizing vacuum filtration, achieving heightened sensitivity in the detection of pesticide residues on alimentary surfaces [126]. In another investigation, Li et al. formulated BCNF–zein-nanoparticle composite films embedded with erythromycin, which exhibited remarkable tensile strength (119 MPa), superior thermal stability, and antibacterial efficacy with inhibition zones of 35 mm against *S. aureus* [127].

Moreover, this technological approach facilitates the creation of multilayer films with customizable architectures through sequential filtration processes. Liu et al. engineered BC/MXene/Hollow Fe_3_O_4_ multilayer composites characterized by dual-gradient electromagnetic structures that enhanced crack resistance and mechanical robustness. To alleviate phase separation induced by incompatible constituents, Pickering emulsions can effectively stabilize the oil–water interface. Miao et al. developed curcumin-loaded Pickering emulsions utilizing protein/polysaccharide hybrid nanoparticles and subsequently integrated them into BC via vacuum filtration, resulting in BC–PE–Cur films with antibacterial properties and pH-responsive indicators, thus suitable for the assessment of food freshness [128].

Thus, vacuum filtration emerges as a proficient and precise fabrication method that circumvents thermal degradation while providing meticulous structural control through the modification of filler composition, filtration sequence, and membrane porosity, thereby facilitating the design of high-performance structural composites [125,126,127,128,129,130,131].

Electrospinning represents another extensively employed technique for the fabrication of polymer nanofibres under high-voltage electrostatic fields. This process is distinguished by its mild preparation conditions, cost-effectiveness, and operational simplicity. The apparatus comprises a syringe equipped with a metallic needle, a syringe pump, a high-voltage power supply, and a metallic collector. In the presence of the electric field, polymer droplets form a Taylor cone at the needle apex, resulting in the ejection of charged jets that solidify into nanofibres upon contact with the collector, culminating in materials characterized by high porosity, extensive surface area, and diverse functionalities [125,129].

Nevertheless, the pronounced intra- and intermolecular hydrogen bonding present in BC constrains its solubility in traditional solvents. Ionic liquids, which are organic salts characterized by melting points below 100 °C, possess the capability to solubilize BC via distinct ionic interactions. Azimi et al. [125] utilized 1-butyl-3-methylimidazolium acetate as a solvent to synthesize electrospun BC nanofibers that exhibit enhanced uniformity, porosity, and water retention capabilities. Despite the general tendency of electrospun fibers to demonstrate suboptimal mechanical strength, it is possible to achieve reinforcement through the incorporation of rigid nanomaterials. Chen et al. [129] harnessed 1-allyl-3-methylimidazolium chloride as a solvent to incorporate multi-walled carbon nanotubes into electrospun BC, resulting in an increase in tensile strength and modulus by 290% and 280%, respectively, in comparison to pristine BC. Furthermore, BC can function as a reinforcing filler within polymer matrices. In contrast to casting and vacuum filtration techniques, electrospinning mitigates the brittleness and restricted porosity associated with BC-based films and facilitates precise control over fiber orientation, pore architecture, and multilayer configurations through the manipulation of spinning parameters. This adaptability lays a robust groundwork for the innovation of advanced high-performance packaging materials derived from BC.

### 5.4. Chemical Functionalization of Bacterial Cellulose for Food-Contact Packaging

Chemical functionalization represents an essential route for tailoring the surface chemistry and physicochemical behavior of BC toward specific food-contact requirements. Unlike physical blending or coating, functionalization involves covalent derivatization of hydroxyl groups on cellulose chains, generating materials with tunable hydrophobicity, reactivity, and barrier performance. Various studies have demonstrated that chemical modification can enhance compatibility with biopolymers, improve film-forming ability, and stabilize mechanical strength under humid conditions [19,21,89]. For instance, acetylation and esterification introduce hydrophobic acetate moieties that reduce water uptake and gas permeability, while oxidation processes introduce carboxyl and aldehyde groups that enable subsequent coupling with active molecules [106,112]. Consequently, chemical functionalization provides a versatile means to integrate durability and bioactivity within sustainable BC-based packaging.

Among the most widely explored approaches are acylation and oxidation reactions, which modify cellulose polarity and surface energy. Acetylation using acetic anhydride or vinyl acetate partially substitutes hydroxyls with acetyl groups, forming cellulose acetate-like structures that improve moisture resistance and transparency [19,21,106]. Oxidation via TEMPO (2,2,6,6-tetramethylpiperidine-1-oxyl) selectively converts primary hydroxyl groups into carboxylates, creating negatively charged sites suitable for ion crosslinking and further grafting with cationic polysaccharides such as chitosan or ε-polylysine [81,124]. These reactions not only increase interfacial adhesion in multilayer composites but also strengthen antimicrobial potential, which is crucial for extending food shelf life. Furthermore, phosphorylation and silylation introduce ionic or organosilicon functionalities that contribute to thermal stability, flame retardancy, and improved water vapor barrier properties [55,89].

Another emerging strategy is cyclic anhydride modification, in which reagents such as succinic or maleic anhydride react with cellulose hydroxyls to form ester linkages that lower crystallinity and enhance flexibility. Jiang et al. prepared strong BC films via cyclic-anhydride modification that exhibited high barrier performance and mechanical strength suitable for active packaging [106]. The introduction of carboxylic groups through this route also promotes compatibility with polyols, waxes, or biobased coatings, thereby reducing delamination under humid storage. Similarly, carbamation with isocyanates generates urethane linkages that provide elasticity and toughness, whereas silanization with alkoxysilanes imparts moisture resistance and improved optical clarity [89,91]. Through careful control of reaction parameters such as reagent concentration, temperature, and degree of substitution, these chemical pathways allow precise tuning of BC film properties for food applications.

Functionalization also supports the immobilization of bioactive compounds, resulting in packaging that combines mechanical integrity with antimicrobial or antioxidant activity. Oxidized or carboxylated BC matrices can covalently bind phenolic acids, catechols, or natural antioxidants, creating active films that inhibit microbial growth without releasing harmful substances [112,128]. For example, TEMPO-oxidized BC crosslinked with chitosan or polylysine demonstrates broad-spectrum antibacterial effects and improved flexibility, while curcumin-embedded emulsions incorporated into modified BC films display enhanced light stability and food preservation efficacy (Table 4) [81,128]. In this context, chemical functionalization acts as a bridge between structural reinforcement and functional activation, ensuring both barrier efficiency and food safety. Moreover, surface grafting facilitates homogeneous dispersion of nanoparticles or natural fillers, which further extends BC’s versatility as a bio-based packaging substrate [91,114].

Since the functionalization approach directly influences composite structure and performance, it is important to illustrate how different modifying-agent classes affect the advantages, limitations, and potential applications of BC-based composites. These relationships are summarized in Table 5, which provides a comparative overview of performance trade-offs relevant to food-packaging design.

Overall, chemical functionalization provides a powerful complement to physical composite formation by enabling molecular-level control of BC’s interfacial and bulk properties. Covalent derivatization techniques such as acetylation, oxidation, phosphorylation, silylation, and anhydride coupling have been successfully applied to achieve improved hydrophobicity, mechanical stability, and bioactivity using non-toxic reagents compliant with food-contact standards [19,55,81,88]. The balance between degree of substitution, crystallinity, and permeability determines the optimal performance of modified films. Overall, the integration of chemical modification into BC processing not only enhances sustainability but also aligns with industrial demands for biodegradable, safe, and multifunctional materials capable of replacing conventional plastics in modern food packaging systems.

## 6. Applications of BC-Based Food Packaging

Food packaging systems establish controlled microenvironments that mitigate external stressors, thereby delaying food deterioration. This protective role is vital for preserving sensory attributes, nutritional integrity, and microbial safety during shelf life. In this context, BC serves as a versatile biomaterial combining controllable porosity, tunable elasticity, and natural biodegradability, enabling its adaptation to various preservation and barrier requirements. Through targeted functionalization, BC can acquire enhanced mechanical, antimicrobial, or antioxidant properties, broadening its suitability for next-generation sustainable packaging applications [132]. Importantly, adoption in commodity formats depends on unit economics at scale, where materials must meet cost-per-area targets and run on existing converting lines without productivity loss; therefore, technical performance must be paired with credible manufacturing routes that lower cost relative to incumbent polyethylene and polypropylene [1,20,21,22].

Beyond laboratory and pilot-scale demonstrations, the transition of BC-based packaging from research to industrial implementation remains constrained by several practical and regulatory challenges. At the industrial level, large-scale fermentation faces high substrate costs, limited reactor oxygen transfer efficiency, and batch-to-batch variability that influence yield and film uniformity. Continuous bioreactor designs and strain engineering are being explored to mitigate these bottlenecks. From a regulatory perspective, BC-based food contact materials must comply with safety frameworks such as the U.S. Food and Drug Administration 21 CFR and the European Union Regulation No 1935/2004, which require comprehensive assessment of migration, biodegradability, and potential additives or nanocomposites used for functionalization. The absence of harmonized global standards for bio-based packaging further complicates commercialization. Moreover, industrial adoption depends on cost-competitiveness with petroleum-derived plastics, scalability of purification and drying steps, and compatibility with existing converting and sealing technologies. Addressing these challenges through integrated techno-economic analysis, green manufacturing, and clear regulatory guidance will be pivotal for the widespread deployment of BC-based food packaging systems [18,19,20,21,78]. In economic terms, media and nutrients can account for a large share of operating costs, which motivates low-cost feedstocks and process intensification; waste-stream substrates and optimized bioprocess conditions reduce raw-material burden and improve yields, directly improving cost-per-kilogram of BC [43,44,45,71,72,79,80,81,82,83,84]. Likewise, energy-intensive purification and drying are significant contributors to cost; process choices that enable continuous production, reduced washing loads, or reel-to-reel dewatering are therefore central to feasibility [44,45,46,96]. Life-cycle assessments indicate that process electricity and chemical inputs dominate environmental and cost hotspots, so improvements that lower energy and solvent use strengthen both sustainability and competitiveness [28,29].

Head-to-head with low-cost plastics, BC will not compete purely as an undifferentiated commodity film; instead, it competes where performance creates value that outweighs material cost. Barrier and mechanical enhancements achieved by chemical functionalization and multilayer constructions improve water-vapor and grease resistance while preserving strength, enabling down-gauging and reduced product loss [109,117,119]. For smart packaging, incremental costs arise from indicators, bioactives, or antimicrobial layers; however, these premiums can be offset when films demonstrably extend shelf life, reduce returns, and cut food waste, which are substantial cost drivers in cold-chain logistics [1,19,22,29]. Consequently, the relevant metric is total cost of ownership rather than resin price alone; in use cases with high spoilage risk or premium produce, BC-based active or intelligent formats can be economically advantageous even if material unit cost exceeds polyethylene [1,19,20,21,22,28]. Moreover, functional layers can be applied at low coat weights and integrated into standard coating, printing, and lamination workflows, which limits capital expenditure and preserves line speeds [106,112,114].

The suitability of BNC for specific food categories depends primarily on its moisture sensitivity, mechanical stability, and response to freeze–thaw conditions. Products with high spoilage rates and moderate moisture levels, such as berries, leafy greens, and fresh herbs, benefit from BNC’s humidity-regulating properties and its capacity to incorporate antimicrobial or antioxidant agents (Table 6) [106]. Dry foods, including cereals and snacks, are also compatible because modified BNC films provide adequate grease and oxygen barriers. In contrast, applications involving frozen foods remain challenging because BNC films undergo structural collapse when subjected to repeated freeze–thaw cycles [110]. High-liquid foods, such as soups or sauces, require sealing integrity and water resistance beyond the typical performance of unmodified BNC. These distinctions reflect material behavior rather than speculative performance claims and indicate where BNC can be realistically deployed within current technological constraints [115].

Traditional food packaging primarily serves as static barriers, while contemporary active packaging systems interact dynamically with food, facilitating the regulated release or absorption of compounds that enhance quality during storage [133,134,135,136,137,138,139,140]. This advancement transitions packaging from mere containment to active quality preservation, enhancing antibacterial and antioxidant efficacy and enabling real-time freshness monitoring [134]. However, polysaccharide- and protein-based materials often exhibit limitations in scalability owing to mechanical deficiencies [136]. Conversely, BC, derived from biosynthesis, features a robust crystalline nanonetwork, making it a viable sustainable reinforcement material [135]. For instance, the incorporation of BC nanowhiskers significantly improves film strength [137]. Additionally, BC–citrus pectin/thyme essential oil composites preserve the structural integrity of BC while achieving remarkable tensile strength and superior moisture barrier properties, effectively maintaining grape quality for 9 d through synergistic reinforcement and hydrophobic modifications [138]. From an implementation perspective, active layers can be metered at grams per square meter using industrially familiar coating or extrusion-lamination steps, which constrains added cost while delivering shelf-life gains that improve retailer margins [106,112,114].

In addition to mechanical strength, packaging materials must also inhibit oxidative spoilage and microbial contamination. Natural antioxidants, including polyphenols and curcumin, are frequently integrated into active films due to their safety and compatibility. Shi et al. created BC–gallic acid composites with notable antioxidant capabilities, significantly extending strawberry shelf life at room temperature due to the phenolic hydroxyl groups in gallic acid that neutralize reactive oxygen species [34]. Agricultural by-products rich in natural antioxidants can further reduce production expenses and foster circular economy initiatives. For instance, Cazón et al. developed BC/chitosan films containing grape bagasse extract, achieving a high phenolic content and effective radical scavenging, thereby safeguarding food from oxidative deterioration [60]. Furthermore, the structural engineering of BC-based composites can improve synergistic functionality. Zhang et al. developed multilayer BC/gellan gum/quaternary ammonium chitosan microsphere films that offered water vapor barrier properties and over 90% antibacterial efficacy against *E. coli* and *S. aureus*. Preservation studies demonstrated that strawberries coated with these multilayer films retained freshness and hydration for up to 5 d [126]. Economically, using waste-derived phenolic sources and low-add-on multilayers reduces formulation cost, while demonstrated extensions in shelf life create measurable value in high-loss categories such as berries and leafy produce [18,19,20,21,22,28,60,126].

Intelligent packaging systems have emerged as a sophisticated category of materials proficient in visually monitoring the freshness of food items through their responses to variations in pH levels, gaseous emissions, and other environmental stimuli. Anthocyanins, curcumin, alizarin, and betaine represent prominent natural pH-sensitive dyes utilized as indicators of freshness. Anthocyanins undergo notable chromatic transitions from red to blue as pH levels increase, thereby reflecting their structural transformation from the flavylium cation to the quinonoid base. Li et al. [127] incorporated anthocyanins into bacterial cellulose-based Pickering emulsions stabilized with camellia oil, resulting in packaging films that transitioned from purple-red to brown as the spoilage of shrimp advanced. Similarly, curcumin demonstrates pH-dependent color alterations linked to keto-enol tautomerism, transitioning from yellow to reddish-brown at elevated pH levels. Miao et al. [128] integrated curcumin into bacterial cellulose to fabricate films that shifted from light to dark yellow as basa fish deteriorated, effectively signaling spoilage and surpassing traditional plastic packaging in efficacy. Furthermore, multicomponent pigment systems have exhibited enhanced sensitivity in comparison to single dye systems. Zhou et al. [139] embedded a curcumin–anthocyanin composite into bacterial cellulose nanofiber/gelatin films that transitioned from yellow to red across a range of acidity levels, indicating significant potential for practical applications in freshness monitoring. From a cost standpoint, most indicator chemistries are low-mass add-ons printable by flexographic or gravure methods, so the dominant expense is integration into converting rather than pigment cost, which supports economic feasibility when indicators prevent out-of-date write-offs [1,19,21,127,128].

Gas-responsive indicator films derived from BC now consistently convey the presence of spoilage gases such as ammonia (NH_3_) and hydrogen sulfide (H_2_S) through pronounced colorimetric and optical alterations. Colorimetric systems that incorporate transition-metal complexes or metal nanoparticles integrated within BC or BCNC demonstrate swift, perceptible reactions to amines and sulfides at concentrations pertinent to food safety [140,141,142,143,144]. For instance, a BC/CMC film that is functionalized with the imidazolium copper complex HIm_2_CuCl_4_ exhibits a reliable transformation from a stable lime-green to blue upon exposure to ammonia in the parts per million (ppm) range, thereby serving as an effective monitoring tool for fish spoilage [141]. Furthermore, matrices of BC/BCNC modified with silver or copper nanoparticles also exhibit darkening in response to ammonia and hydrogen sulfide due to redox or electronic transitions of the metallic constituents, while silver nanoparticles embedded in BC nanopaper can be influenced by gaseous ammonia—mechanisms that provide a robust basis for effective visual sensing [140,142,143]. Because sensing layers can be deposited by low-cost printing and slot-die coating at minimal coat weights, the marginal cost per package remains small relative to avoided spoilage and improved quality assurance [114,126,127,128,140,141,142,143].

Beyond cinematic productions, edible and functional coatings present significant appeal due to their ability to be applied via spraying or dipping methodologies, thereby ensuring uniform coverage of irregularly shaped food items, regulating gas and moisture exchange, and facilitating the delivery of bioactive compounds. Pickering-emulsion systems, which are stabilized by colloidal biopolymers, permit the controlled release of essential oils that exhibit potent antioxidant and antimicrobial properties; notably, this methodology is seamlessly applicable to coatings reinforced with BC [144]. The integration of BC nanofibers as nanofillers substantially enhances the mechanical strength of gelatin-based edible coatings and contributes to the preservation of fresh-cut apple quality during storage, all while preserving a high degree of visible transparency for visual assessment [145]. To achieve anti-adhesion properties and facilitate the easy removal of contents from packaging, superhydrophobic BC-based coatings that are engineered with inorganic waxy phases and silica roughness effectively reduce residue left by viscous food substances (e.g., honey, yogurt). Scalable systems composed of BC nanosilica and beeswax have demonstrated water contact and sliding angles of approximately 153° and 3°, respectively, thereby providing self-cleaning and anti-fouling capabilities that are well-suited for reusable or low-waste packaging formats [146]. Critically, these coatings are applied at low thickness and can be processed on standard lines, which lowers the incremental cost and eases competition with polyethylene coatings in niche, performance-driven formats [1,20,21,22,114,146].

Hydrogel and aerogel configurations derived from BC expand their applicability from moisture and odor regulation to cushioning and proactive protection. Moisture-absorbing BC–guar-gum hydrogels enhance barrier and mechanical properties for berry packaging while effectively managing headspace humidity [147]. Pertaining to ethylene regulation, BC hydrogels integrated with microalgae serve as biologically based ethylene scavengers, achieving over 90% removal efficiency, thereby prolonging the shelf life of fruits and vegetables [148]. Additionally, an innovative active-packaging strategy involves the creation of oxygen-scavenging coatings through the immobilization of enzymes (such as glucose oxidase) or redox agents within cellulosic matrices, a methodology that is compatible with BC substrates [149]. In the context of pads and liners, BC-reinforced antibacterial aerogels (for instance, CMC@AgNP/BC/citric-acid) synergistically combine rapid exudate absorption with robust antimicrobial properties, significantly reducing bacterial load and color degradation of chilled meats over a seven-day period [150]. Ultimately, foam-templated porous BC films and aerogels provide adjustable pore size and thickness under ambient conditions and can be subsequently functionalized (for example, with chitosan) to enhance liquid absorption and antibacterial efficacy while preserving low density and cushioning capabilities [151,152]. Because pads, liners, and inserts are used at very low mass per pack, these formats are among the earliest economically feasible BC applications, where performance benefits justify modest material premiums [21,22,23,28,150,151,152].

Material cost remains one of the main constraints for the wider deployment of BNC-based food packaging and is strongly shaped by process conditions rather than chemistry alone [97,110]. Pilot- and industrial-scale studies show that expenses associated with carbon sources, fermentation time, and downstream operations such as purification and drying significantly elevate the production cost of BNC compared with commodity thermoplastics [96,97,98,99]. Nevertheless, several works demonstrate that using organic residues and low-value side streams as feedstocks, together with in situ composite formation, can lower medium costs and improve space–time yields, which directly reduces the effective cost per kilogram of BNC [41,114,115,116,117,118,119,120,121,122,123,124,125,126,127]. Advanced structuring strategies, including force-induced alignment, nacre-inspired architectures, and multilayer films, achieve mechanical and barrier properties that are competitive with or superior to conventional plastics, allowing thinner gauges or multifunctional formats that can partially offset higher material prices in demanding applications [98,106,108,114,119]. Overall, current evidence indicates that BNC occupies a higher-cost regime than polyethylene and polypropylene and is therefore best suited for packaging formats where performance gains, added functionality, or sustainability drivers can justify a higher material price (Table 7).

Overall, the economic feasibility of BC-based and smart packaging improves when processes exploit low-cost feedstocks, continuous or intensified operations, and thin functional layers integrated on incumbent equipment [43,44,45,71,72,79,80,81,82,83,84,95,106,112,114]. Competition with polyethylene is realistic in segments where waste reduction, product quality, brand sustainability claims, or regulatory drivers provide measurable value that offsets higher material cost [1,19,20,21,22,27,28,106,112,114]. Consequently, BC is best positioned for high-value produce, protein, and premium ready-to-eat categories, as well as function-specific components such as pads, labels, and intelligent indicators that leverage minimal coat weight with maximum effect [150,151,152,153].

## 7. Challenges and Future Work

Despite remarkable progress in developing BC-based bioplastics for food packaging, several persistent challenges continue to limit their industrial scalability, environmental resilience, and cost efficiency [60]. Despite its advantageous optical clarity, structural cohesion, and capacity for biological decomposition relative to other biopolymers, its transition from laboratory production to commercial implementation remains hindered by both technological and regulatory constraints. Recent advances have improved BC performance through functionalization and structural tuning [97,110], yet these improvements have not fully addressed the systemic barriers associated with scale-up, cost, and long-term stability.

One of the central obstacles involves the modification of BC with exogenous additives such as nanomaterials, carbon-based compounds, and plasticizers that enhance its strength, flexibility, or barrier properties. While these modifications improve functional performance, their long-term environmental and health impacts remain uncertain [77]. The potential migration of nanoparticles or plasticizers into food products poses biosafety risks, highlighting the urgent need for internationally harmonized standards and regulations. Evidence from active and intelligent packaging research demonstrates that certain nanoparticles, essential oils, and responsive dyes exhibit concentration-dependent migration behavior [133,134,135,136,137,138,139,140,141,142,143], reinforcing the necessity for standardized toxicological and migration testing (e.g., EFSA, FDA). Future studies should focus on defining safe concentration limits for additives, conducting systematic toxicological assessments, and performing full life-cycle analyses to ensure environmental compliance and consumer safety.

From an economic standpoint, BC production remains costly due to the price of conventional culture media and the length of the fermentation process [127]. Achieving large-scale feasibility requires optimization of both substrates and process conditions, including the selection of well-defined biomass feedstocks for fermentation. The substitution of refined carbon sources with agricultural or industrial residues such as fruit waste, molasses, or crude glycerol, that is, lignocellulosic and carbohydrate-rich biomass streams that serve as low-cost carbon sources for BC-producing strains, can significantly reduce production costs while promoting sustainability. Additionally, the adoption of open or semi-continuous fermentation systems could minimize energy consumption and shorten production time. Advances in metabolic engineering and synthetic biology may further improve cellulose biosynthesis by redirecting metabolic fluxes and enhancing substrate utilization efficiency. Directed evolution and strain optimization approaches have recently proven effective in increasing productivity and modifying fibril architecture [122], offering promising tools for cost reduction.

Another limitation concerns the relatively rapid biodegradation of BC under natural environmental conditions. The high density of hydroxyl groups promotes enzymatic hydrolysis and leads to complete degradation within roughly six months [150]. Although this property supports ecological sustainability, it compromises the structural stability required for packaging high-moisture foods including dairy, fresh produce, and chilled meats, and it becomes even more critical for frozen products, where ice-crystal formation and freeze–thaw cycles can induce irreversible structural collapse of BNC films. Freeze–thaw instability has been consistently reported in studies evaluating cold-chain conditions, indicating that BNC cannot maintain dimensional or mechanical integrity during frozen storage [106]. Achieving equilibrium between environmental degradability and mechanical durability is therefore essential for applications spanning chilled and frozen food packaging. Techniques such as mild cross-linking with biocompatible agents, surface hydrophobization, or the design of multilayered composites could extend the material’s functional lifespan without compromising its eco-friendly profile.

In addition, the limited flexibility, ductility, and puncture resistance of BC restrict its use in demanding packaging applications [107]. These issues can be mitigated by bioinspired structural design that imitates natural composites, by incorporating flexible matrices such as gelatin, polycaprolactone, or thermoplastic starch, and by controlling fibril alignment during biosynthesis to achieve desired mechanical orientation. The inclusion of reinforcing nanofillers such as lignin, cellulose nanocrystals, or graphene oxide may further enhance strength while preserving biodegradability and transparency. Emerging approaches such as nacre-inspired hybrid films, ultrathin BC layers, and mica-reinforced nanostructures have demonstrated substantial gains in tensile strength and dimensional stability [106,107,109,119], highlighting the importance of hierarchical design strategies.

Current research on BC packaging remains concentrated on perishable items such as fruits, vegetables, and meat. Broader exploration of its suitability for processed, frozen, and ready-to-eat products could expand its commercial reach. Future innovations should also focus on the integration of smart functions including pH indicators, spoilage sensors, and digital tags to create intelligent BC-based packaging capable of real-time monitoring and traceability throughout the food supply chain.

In addition to technological and economic considerations, future progress critically depends on the establishment of standardized shelf-life testing protocols for BC-based composites. Presently, most reported performance data are derived from small-scale laboratory trials under controlled conditions, which often fail to replicate the complexity of real food matrices. Large-scale, controlled studies involving perishable commodities such as meat, cheese, and fresh produce are essential to accurately assess barrier stability and microbial resistance under realistic storage and handling environments. Such studies should quantitatively evaluate parameters including oxygen transmission rate, water vapor transmission rate, and microbial spoilage kinetics relative to benchmark petroleum-based plastics. Harmonized methodologies will enable meaningful cross-comparison of results, support regulatory acceptance, and provide industry with reliable indicators of performance and shelf-life extension potential.

Moreover, given the incorporation of novel nanomaterials, cross-linkers, and plasticizers in BC functionalization, regulatory evaluation must accompany technical development. The current and anticipated regulatory frameworks established by the U.S. Food and Drug Administration and the European Food Safety Authority categorize many nanomaterial- and plasticizer-based additives as food-contact substances that require migration, toxicological, and environmental assessments prior to approval. Additives such as silver nanoparticles, polyethylene glycol, citric acid, and polycaprolactone exhibit varying authorization statuses depending on concentration and intended use, underscoring the need for continuous alignment between formulation strategies and evolving safety standards. Clear documentation of compliance pathways and harmonized global guidelines will be critical for ensuring consumer safety, facilitating market entry, and accelerating the industrial translation of BC-based food packaging systems.

Ultimately, the advancement of BC-based packaging requires a multidisciplinary approach that combines material science, biotechnology, and circular-economy principles. Priority should be given to the development of scalable fermentation systems, cost-effective medium formulations, and verified biosafety protocols. When coupled with intelligent sensing technologies and optimized mechanical design, BC has the potential to evolve from a research material into a practical and sustainable alternative to petroleum-based plastics for modern food packaging applications. Although this review provides a comprehensive overview of the progress and prospects of bacterial cellulose–based food packaging, it has several inherent limitations. The discussion relies primarily on peer-reviewed literature available in English and does not encompass patent data or unpublished industrial reports that could further clarify large-scale implementation trends. Quantitative comparison of production yields, economic costs, and environmental impacts across different studies remains challenging because of variability in experimental designs and reporting standards. Therefore, future systematic meta-analyses and techno-economic assessments are needed to complement this review and strengthen the evidence base for industrial translation.

## 8. Conclusions

BC represents a sustainable, renewable, and environmentally friendly material with strong potential to replace petroleum-derived plastics. However, its limited antibacterial, antioxidant, and barrier properties still hinder industrial scalability, prompting continued efforts to improve functionality through in situ and ex situ modification strategies involving the incorporation of diverse exogenous compounds.

In in situ methodologies, additives are integrated directly into the BC matrix during the cultivation phase via modified nutrient formulations, aerosol-assisted biosynthesis, or three-dimensional biofabrication. Conversely, ex situ techniques such as impregnation, casting, vacuum filtration, and electrospinning involve the incorporation of functional components subsequent to BC synthesis, which affords enhanced control over composition and structural characteristics. These modification methodologies enhance antibacterial, antioxidant, and barrier properties, thereby enabling BC to impede microbial proliferation, regulate gas permeability, manage temperature and humidity, and provide ultraviolet protection. Moreover, the incorporation of responsive molecules has paved the way for the development of intelligent packaging systems capable of detecting spoilage or environmental fluctuations, in addition to thermal management packaging that preserves food quality under variable storage conditions.

Despite considerable advancements in the functionalization and application of BC-based packaging, several challenges persist unaddressed. Technical impediments associated with large-scale production, economic viability, environmental compatibility, and the lack of consistent regulatory frameworks continue to hinder its commercialization. Future inquiries should therefore concentrate on optimizing production efficiency, reducing manufacturing costs, and undertaking extensive environmental and toxicological evaluations to guarantee product safety. Once these obstacles are effectively surmounted, BC-based bioplastics could potentially emerge as a revolutionary and sustainable substitute for synthetic plastics, providing substantial environmental and societal advantages for subsequent generations.

## Figures and Tables

**Table 1:** Comparison of static and agitated BC production modes [38,39,40,41,42].

Criterion	Static Culture	Agitated Culture
Typical strains (examples)	*K. xylinus*, *K. hansenii*	*K. xylinus* (agitation-tolerant variants), *K. rhaeticus*
Culture type/vessel	Trays or shallow flasks; air–liquid interface	Shaken flasks or stirred-tank bioreactors
Product morphology	Continuous pellicle films	Pellets/irregular particles; sometimes spherical aggregates
Fermentation duration	~7–14 d at 28–30 °C	~2–5 d at 28–30 °C (higher O_2_ transfer)
Cellulose yield (indicative)	Up to ~20 g L^−1^ under optimized media	Often lower per batch volume but higher volumetric productivity due to shorter cycles
Purity/crystallinity (qual.)	High crystallinity; high CI; long DP	Moderate crystallinity; shorter DP; more defects
Scalability	Limited by oxygen diffusion and manual handling	Good; compatible with fed-batch and controlled aeration
Downstream processing	Minimal purification for pellicles	Additional classification if uniform film is needed
Cost considerations	Lower CAPEX; higher labor and longer cycle time	Higher CAPEX; better throughput; sensitive to strain instability
Common risks	Surface contamination; O_2_ limitation	Mutation to non-producers under shear; lower film quality

Here, CI = crystallinity index; DP = degree of polymerization.

**Table 2:** Key properties and performance of BC.

Preparation Method	Fabrication Technique/Category	BC Form/Morphology	Composition	Key Performance Characteristics	Enhanced Functional Property	Application Field	Ref.
In situ modification							
Modified medium cultivation	Biosynthesis	Hydrogel membrane	BC/gellan gum	Superior oxygen barrier efficiency (57.6 cm^3^ day^−1^ m^−2^).	Improved gas impermeability	Food packaging	[115]
	Biosynthesis	Membrane film	BC/xanthan gum	Strong FTIR band at 1323 cm^−1^; tensile strength 40 MPa (dry) and 6.8 MPa (wet).	Enhanced mechanical strength	Packaging films	[116]
	Biosynthesis	Nanofibrillar mat	BC/mulberry leaves	High antibacterial efficacy (99%) against *E. coli* and *S. aureus*.	Antimicrobial activity	Active food coating	[117]
	Biosynthesis	Nanofibrous film	TIPEG–Glucose–BC	Dual antibacterial activity: 99% (*E. coli*), 95% (*S. aureus*).	Biocidal surface functionality	Biomedical dressings	[118]
	Aerosol-assisted biosynthesis	Dense membrane	BC/synthetic mica	High tensile strength (375 MPa); toughness 25.9 MJ m^−3^; elongation 2.9%; UV blocking 92%.	Mechanical reinforcement and UV shielding	Flexible packaging	[109]
	Aerosol-assisted biosynthesis	Nanofibrous mat	BC/nano-clay	Strength 63.5 MPa; modulus 2.7 GPa; elongation 2.9%; gas barrier improved by 27%.	Mechanical and barrier enhancement	Packaging and coatings	[119]
	Aerosol-assisted biosynthesis	Nanocomposite film	BC/SiO_2_	Strength 35.5 MPa; modulus 3.0 GPa; antibacterial efficiency 95.4%.	Hybrid reinforcement with antibacterial effect	Biomedical and packaging	[120]
	Aerosol-assisted biosynthesis	Conductive network	BC/AgNW	Electrical conductivity 5.2 S cm^−1^; mechanical strength 5.8 MPa.	Conductive functionality	Flexible electronics	[121]
	Aerosol-assisted biosynthesis	Porous membrane	BC/gellan gum + pomegranate microspheres	Antibacterial rate 99%; tensile strength 6.2 MPa; elongation 14.7%; barrier efficiency at 63% RH.	Antimicrobial and flexible performance	Food preservation	[119]
3D biofabrication	3D bioprinting	Hydrogel ink	BC/κ-carrageenan	Suitable rheology for 3D printing with stable structural fidelity.	Enhanced printability	Tissue engineering	[122]
	Molding	Molded film	BC	Shape precision in negative-mold forming.	Dimensional stability	Biomedical scaffolds	[123]
	Bio-printing	Biocomposite hydrogel	BC/CS	Antibacterial activity (99% *E. coli*); tensile strength 9 MPa; modulus 2.43 GPa.	Strength and antibacterial performance	Biomedical implants	[124]
*Ex situ* modification							
Casting	Solvent casting	Film	BCNP + Curcumin + Cinnamon oil	UV shielding and antibacterial efficiency 99% (*S. aureus*).	UV barrier and antimicrobial functionality	Food packaging	[111]
	Solvent casting	Film	BCNP + Polylactic + Ferulic acid	Strength increase 40%; modulus increase 68%.	Enhanced mechanical reinforcement	Bioplastic replacement	[110]
	Solvent casting	Composite film	BCNP + Zn^2+^ + Sodium alginate	Antibacterial activity 98.1% (*E. coli*, *S. aureus*); tensile strength 47 MPa.	Antimicrobial and structural durability	Active packaging	[114]
	Solvent casting	Flexible film	BCNP + Chitosan	Strength 47.2 MPa; improved gas-barrier capacity.	Barrier integrity and toughness	Biodegradable wraps	[125]
Vacuum filtration	Filtration assembly	Nanofibrous film	BCNP + Ag nanoparticles	Disinfection rate ≈10^4^ CFU mL^−1^ reduction; strength 35 MPa.	Antimicrobial and durable surface	Water purification	[126]
	Filtration assembly	Porous sheet	BCNP + Zn-doped hydroxyapatite	Strength 37.4 MPa; water contact angle ≈ 106°.	Hydrophobic and antibacterial surface	Biomedical coatings	[127]
	Filtration assembly	Composite sheet	BCNP + Pickering emulsions	Strength 24.5 MPa; antibacterial efficiency > 98%.	Antimicrobial and dispersion uniformity	Smart packaging	[128]
Electrospinning	Electrospinning	Nanofiber mat	BCNP + Chitin nanofibers	Tensile strength 4–5 MPa; modulus 0.8 GPa.	Flexibility and structural strength	Biomedical mesh	[125]
	Electrospinning	Conductive nanofiber	BCNP + Carbon nanotubes	Strength 14 MPa; electrical conductivity 0.45 S cm^−1^.	Electrical conductivity	Flexible electronics	[129]

BC = Bacterial cellulose; BCNP = Bacterial cellulose nanofibrils; TIPEG = Chitosan photo-initiated ethylene glycol; AgNW = Silver nanowire; CS = Chitosan; RH = Relative humidity; UV = Ultraviolet.

**Table 3:** Comparison of mechanical and thermal properties of BC/BNC and traditional petroleum-based plastics.

Material	Tensile Strength (MPa)	Young’s Modulus (GPa)	Toughness (MJ m^−3^)	Thermal Stability (Tm/Td)	Refs.
Bacterial cellulose (BC)	18–22	15–18	–	Tdmax 319–374 °C	[47,62,106,108]
Ultrathin drawn BC films	758	–	42	Stable up to ~300 °C	[71,107]
BC nanocomposites (e.g., BC/SiO_2_)	35–63	2.7–3.0	–	Thermally stable up to ~250 °C	[119,120]
Polyethylene (PE)	20–40	0.2–1.0	1–2	Softening ~80–110 °C	[112]
Polypropylene (PP)	30–40	1.0–2.0	~2	Softening ~100–140 °C	[1,20,21,22]
Polycarbonate (PC)	55–75	2.0–2.4	3–4	Softening ~150 °C	[1,20,21,22]
Polyamide (Nylon-6)	50–80	1.5–2.0	2–3	Melting ~220 °C	[68,72]

**Table 4:** Representative chemical agents used for BC functionalization.

Functionalization Agent	Chemical Formula	Reaction Type	Primary Effect on BC	Functionalization Agent	Refs.
Acetic anhydride	C_4_H_6_O_3_	Acetylation	Increases hydrophobicity, reduces water uptake	Acetic anhydride	[19,21]
Vinyl acetate	C_4_H_6_O_2_	Acetylation	Improves transparency and moisture resistance	Vinyl acetate	[19,21,106]
Succinic anhydride	C_4_H_4_O_3_	Cyclic anhydride esterification	Enhances flexibility and barrier stability	Succinic anhydride	[20,21]
Maleic anhydride	C_4_H_2_O_3_	Cyclic anhydride esterification	Improves adhesion and introduces carboxyl groups	Maleic anhydride	[106]
TEMPO	C_9_H_18_NO •	Oxidation catalyst	Produces carboxylate-rich BC for grafting	TEMPO	[81,128]
Sodium hypochlorite (NaClO)	NaClO	TEMPO co-oxidant	Facilitates selective oxidation of primary hydroxyls	Sodium hypochlorite (NaClO)	[19,21,49]
Isocyanates (e.g., HDI)	O=C=N–R–N=C=O	Carbamation	Creates urethane linkages and increases toughness	Isocyanates (e.g., HDI)	[89,91]
Trialkoxysilanes (e.g., TEOS)	Si(OC_2_H_5_)_4_	Silylation	Improves water-vapor barrier and thermal stability	Trialkoxysilanes (e.g., TEOS)	[19,20]
Phosphoric acid	H_3_PO_4_	Phosphorylation	Adds ionic groups and enhances flame retardancy	Phosphoric acid	[19,20]

**Table 5:** Advantages and disadvantages of BC composites.

Modifying-Agent Class	Typical Composite	Advantages	Disadvantages	Suitable Applications	Refs.
Acylation agents	BC-acetate, BC-succinate films	Improved hydrophobicity, better moisture barrier, high transparency	Reduced biodegradation at high substitution	Dry foods, light-sensitive products	[18,20]
Oxidizing agents	TEMPO-BC, dialdehyde BC	Enables grafting, strong antimicrobial potential	Over-oxidation decreases strength	Active antimicrobial packaging	[20,21]
Isocyanates	BC-urethane films	Increased toughness and flexibility	Toxicity concerns during processing	Flexible packaging, pouches	[19,20]
Silanes	Silanized BC nanocomposites	Excellent moisture resistance, better thermal stability	Higher processing cost	High-moisture foods, semi-rigid packaging	[18,19,20]
Phosphorylating agents	Phosphorylated BC	Flame retardancy, ionic conductivity	May increase water affinity	Heat-processed foods	[20,21,22]
Phenolic grafting	BC-gallic acid, BC-catechol	Antioxidant stability, extended shelf life	Possible color changes	Fresh produce	[18,19,20,21,70]
Nanoparticle-assisted	BC-AgNP, BC-Cu^2+^	Antibacterial and barrier enhancement	Regulatory restrictions	Antimicrobial wraps	[18,19,20,21,70]

**Table 6:** Recommended versus not-recommended food categories for BNC packaging.

Food Category	Recommended?	Rationale	Refs.
Fresh berries, cherries	Yes	High spoilage risk; BC extends shelf life through moisture control and antioxidant loading	[106,107]
Leafy greens, herbs	Yes	BC maintains humidity balance and reduces wilting	[21,22,117]
Chilled meats and fish	Yes	BC aerogels absorb exudate and inhibit bacterial growth	[18,56,114]
Dry snacks, cereals	Yes	Modified BC provides good grease and oxygen barriers	[21,71]
Dairy (soft cheeses, yogurt lids)	Conditional	Works if hydrophobic modification applied	[20,111,112,113,114]
Frozen foods	No	Freeze–thaw cycles cause irreversible structural collapse of BNC films	[56,71,106]
High-liquid foods (soups, sauces)	No	Requires high water resistance and seal integrity beyond typical BC capabilities	[21,22,106,107]

**Table 7:** Approximate cost comparison of BNC and conventional plastics [18,98,106,108,114,119].

Material	Approximate Cost (USD/kg)	Cost Per m^2^ of Typical Film	Notes	Refs.
BNC (pilot–industrial scale)	8–15 USD/kg	Higher than PE and PP due to fermentation, purification, and drying requirements	Costs decrease when low-cost feedstocks and continuous or semi-continuous processing are used	[18,98,106]
Polyethylene (LDPE/HDPE)	1–2 USD/kg	Lowest cost per m^2^	Large-scale global production and efficient film-conversion infrastructure	[18,108]
Polypropylene (BOPP/CPP)	1.2–2.5 USD/kg	Low–moderate cost per m^2^	Good moisture barrier and widespread industrial adoption	[114,119]

## Data Availability

The authors confirm that the data supporting the findings of this study are available within the article.

## Abbreviations

The following abbreviations are used in this manuscript:

BC	Bacterial cellulose
HS	Hestrin–Schramm medium
UDP	Uridine diphosphate
TCA	Tricarboxylic acid cycle
DP	Degree of polymerization
WHC	Water-holding capacity
CF	Calcofluor
CMC	Carboxymethyl cellulose
NaOH	Sodium hydroxide
*G. xylinus*	*Gluconacetobacter xylinus*
*K. xylinus*	*Komagataeibacter xylinus*
*G. hansenii*	*Gluconacetobacter hansenii*
*N. hansenii*	*Novacetimonas hansenii*
*A. xylinum*	*Acetobacter xylinum*
I*α*	Triclinic allomorph of cellulose I
I*β*	Monoclinic allomorph of cellulose I
CFU	Colony-forming units
NaClO	Sodium hypochlorite
T_max_	Maximum decomposition temperature
GPa	Gigapascal
MJ m^−3^	Megajoule per cubic meter
°C	Degree Celsius
rpm	Revolutions per minute
pH	Potential of hydrogen
COVID-19	Coronavirus disease 2019
EU	European Union
in situ	Modification performed during biosynthesis/fermentation
ex situ	Modification performed after biosynthesis on purified BC
BCNF	Bacterial cellulose nanofibrils
BCNC	Bacterial cellulose nanocrystals
BCNP	Bacterial cellulose nanoparticles
XG	Xanthan gum
HA	High-acyl (gellan gum)
LA	Low-acyl (gellan gum)
κ-CG	Kappa-carrageenan
PVA	Polyvinyl alcohol
Gly	Glycerol
UV	Ultraviolet
IR	Infrared
PRC	Passive radiative cooling
Bio-RC	BC-based radiative-cooling film
AgNW	Silver nanowire
AgNP	Silver nanoparticles
AgNO_3_	Silver nitrate
NaBH_4_	Sodium borohydride
ZnCl_2_	Zinc chloride
AL	Alkali lignin
CS	Chitosan
MXene	2D transition-metal carbide/nitride
Fe_3_O_4_	Magnetite (iron(II,III) oxide)
SERS	Surface-enhanced Raman scattering
AgNRs@BC	Silver-nanorod-decorated bacterial cellulose
PE	Pickering emulsion
RH	Relative humidity
WCA	Water contact angle
U.S.	United States
TEMPO	2,2,6,6-tetramethylpiperidine-1-oxyl
SCOBY	Symbiotic Culture of Bacteria and Yeast
